# The impact of laparoscopic gynecological surgery training on the technicity index of a developing country center

**DOI:** 10.1590/acb382723

**Published:** 2023-08-21

**Authors:** Daniel Spadoto-Dias, Flávia Neves Bueloni-Dias, Waldir Pereira Modotti, Maria Laura Marconi França, Rodrigo Takeshi Chihara, Pauline Chauvet, Benoit Rabischong, Revaz Botchorishvili, Nicolas Bourdel, Michel Canis

**Affiliations:** 1 Universidade Estadual Paulista “Júlio de Mesquita Filho” – Department of Gynecology and Obstetrics – Botucatu (São Paulo), Brazil.; 2 Institute of Medical Care – Gynecological Endoscopy – Assis (São Paulo), Brazil.; 3 Universidade Estadual Paulista “Júlio de Mesquita Filho” – Department of Gynecology and Obstetrics – Botucatu (São Paulo), Brazil.; 4 University of Clermont Auvergne – Department of Surgical Gynecology – Clermont-Ferrand, France.; 5 University of Clermont Auvergne – Department of Surgical Gynecology – Clermont-Ferrand, France.

**Keywords:** Developing Countries, Education, Medical, Continuing, Hysterectomy, Index, Laparoscopy, Public Health Systems Research

## Abstract

**Purpose:**

To compare laparoscopic gynecological surgery training between a developed country’s reference center (host center) and a public reference service in a developing country (home center), and use the technicity index (TI) to compare outcomes and to determine the impact of laparoscopic gynecological surgery fellowship training on the home center’s TI.

**Methods:**

The impact of training on the home center was assessed by comparing surgical performance before and after training. TI was assessed in 2017 in the host center, and before and after training in the home center. Epidemiological and clinical data, and information on reason for surgery, preoperative images, estimated intraoperative bleeding, operative time, surgical specimen weight, hospital stay length, complication and reintervention rates were collected from both institutions. Home center pre-training data were retrospectively collected between 2010 and 2013, while post-training data were prospectively collected between 2015 and 2017. A two-tail Z-score was used for TI comparison.

**Results:**

The analysis included 366 hysterectomies performed at the host center in 2017, and 663 hysterectomies performed at the home center between 2015 and 2017. TI in the host center was 82.5%, while in the home center it was 6% before training and 22% after training. There were no statistical differences in length of hospital stay, preoperative uterine volume, surgical specimen weight and complication rate between centers. However, significantly shorter mean operative time and lower blood loss during surgery were observed in the host center.

**Conclusions:**

High-quality laparoscopic training in a world-renowned specialized center allowed standardizing laparoscopic hysterectomy procedures and helped to significantly improve TI in the recipient’s center with comparable surgical outcomes.

## Introduction

Laparoscopic gynecological surgery has gained acceptance as a diagnostic and therapeutic tool in the management of several conditions. It decreases postoperative pain and provides shorter hospital stay, faster recovery and better aesthetics without increasing morbidity and complications^1^-^5^. However, although these benefits can together reduce the direct and indirect costs associated with surgery, the open approach remains predominant in many parts of the world. As a matter of fact, laparoscopic training for therapeutic surgeries or even purely diagnostic procedures is not incorporated into the curriculum of a number of gynecology residency programs. In the United States of America, for example, although 73% of the residency programs lead off laparoscopic skill, only 29% of them provide a structured surgical curriculum, and 55% have facilities for training laparoscopy^6^-^8^. Moreover, over 40% of accredited North American Obstetrics and Gynecology residency programs are reportedly dissatisfied with their laparoscopy training^9^.

In Latin America, like much of the developing world, no teaching models for laparoscopic skills or validated tools to evaluate them are commonly available during residency^7^. Among factors that justify the reduced diffusion of laparoscopy, especially in developing countries, are lack of planning and structure within institutions, cost constraints, shortage of qualified teaching personnel, limited resident work hours, and lack of interest due to the inherently difficult learning curve associated with minimally invasive surgical (MIS) approaches, which is longer than the traditional open approach^10^-^14^. The need for using a validated training program that includes performance evaluation before progressing to real procedures has been pointed out by several regulatory bodies^10^. Thus, international laparoscopic gynecological fellowships in experienced centers can offer a good opportunity for knowledge sharing provided that recipients are committed to return to their home countries and disseminate the model learned.

Quality indicators are critical for the assessment of surgical performance. The technicity index (TI) is a straightforward indicator that has been used to compare surgical performance among hospitals. It quantifies the number of minimally invasive hysterectomies over the total number of hysterectomies performed in a single center annually, and may be used as a benchmarking to promote less invasive surgical approaches and improve performance at all levels^15^,^16^. The use of performance indicators can provide reliable data for the evaluation of MIS rates and comparison between centers so that hospital administrators can adequate instrumentation and operating room accessibility^16^.

Considering this framework, the objectives of this study were twofold: first, to compare laparoscopic gynecological surgery training between a developed country’s world-renowned reference center (host center) and a public reference service in a developing country (home center); and secondly, to utilize the TI as a metric to compare professionals, services and surgical outcomes between the host and home centers, in order to determine the impact of comprehensive laparoscopic gynecological surgery fellowship training on the home center’s TI.

## Methods

With the aim of acquiring improved skills for high complexity laparoscopic surgeries, as well as developing a laparoscopic training model for beginner surgeons at their home center, two graduate surgeons from the Department of Gynecology and Obstetrics of Botucatu Medical School (Universidade Estadual Paulista “Júlio de Mesquita Filho”–UNESP, SP, Brazil) participated in a one-year fellowship program in advanced laparoscopic surgical training. The program consisted of half-day activities at both Clermont-Ferrand International Center for Endoscopic Surgery (CICE) and at Clermont-Ferrand Hospital and University Center (CHU) – Estaing (Clermont-Ferrand, Auvergne, France) simultaneously.

Training at CICE consisted of biweekly theory and practice sessions. The theoretical part covered basic and advanced principles of laparoscopic surgery; aspects of the pelvic anatomy and landmarks; indication and application of laparoscopic treatment for pelvic masses, endometriosis, uterine prolapse and cancer; principles of laparoscopic intra- and extracorporeal suturing techniques. Hands-on training was provided using a pig model and box trainers for suturing and other exercises. After three months, trainees started serving as instructors in other classes.

At CHU – Estaing, one-year training included an observational internship of advanced laparoscopic surgeries and participation in real procedures as first assistant. Surgeries included highly complex procedures such as pelvic prolapse repair, surgery for deep infiltrative endometriosis, and pelvic and paraaortic lymphadenectomies in oncologic patients.

In 2014, the newly gained knowledge and skills were shared with the home center. Education materials were translated and adapted to suit the reality of the home center. Theoretical topics including laparoscopic anatomy, laparoscopic instrument handling, entry and insufflation techniques, and principles of electro-surgery were presented in 30-minute lectures to the medical staff of the Gynecological Endoscopy and Family Planning Division of the home center that was thus trained to teach the model learned during fellowship. In 2015, this standardized technique was implemented at the home center on a basis similar to that of the host center, except for the practical animal activity. Monthly theoretical sessions were held, and activities in the inanimate dry laboratory, as well as surgical procedures with second- and third-year Obstetrics and Gynecology residents as first assistants, were systematically implemented within the Gynecological Endoscopy and Family Planning Division.

To evaluate the impact of those activities on the home center, a comparative analysis of surgical performance before and after training was performed. The surgical performance indicator used was the TI. TI was assessed in 2017 in the host center, and before and after training in the home center, according to Eq. 1:


$$\mathrm{TI}=\frac{\text{Total number of minimally invasive hysterectomies}}{\text{Total number of hysterectomies}}\times 100$$ (1)

Hysterectomy was chosen because it is a well-established and standardized technique whose results are reproducible and comparable with a learning curve lower than other procedures of higher complexity^17^,^18^. In both centers, laparoscopic hysterectomy was performed according to standardized surgical steps which are well established and described elsewhere^19^.

Epidemiological and clinical data, as well as information on reason for surgery, preoperative images, estimated intraoperative blood loss, operative time, weight of the surgical specimen, hospital stay length, complication and reintervention rates were collected from both institutions. Complications were defined as any adverse event related to surgery requiring any surgical or nonsurgical reintervention. Home center pre-training data were retrospectively collected between 2010 and 2013, while post-training data were prospectively collected between 2015 and 2017. A two-tail Z-score was used to evaluate TI in each period, with P = 0.05 indicating the level of significance^20^.

For data analysis, measures of location and variability were calculated. Mean, standard deviation, median, and minimum/maximum values for quantitative variables, and absolute frequency and percentage for qualitative variables were estimated. Normally distributed and not normally distributed quantitative variables were compared using the Student’s t-test and the Mann-Whitney’s non-parametric test , respectively^20^. Qualitative variables were analyzed using the Goodman’s test for contrasts among multinomial populations^21^. Data analysis was performed using Statistical Package for the Social Sciences (SPSS) for Windows, version 21.0, with significance level set at 5%. This study was approved by the center’s Committee of Research Ethics under number 29020114.0.0000.5411.

## Results

In order to make our data more comparable, the analysis included 366 hysterectomies performed at the host center (CHU – Estaing) between January and December 2017, and 663 hysterectomies performed at the home center (Botucatu Medical School Hospital/UNESP) between January 2015 and October 2017. At the home center, post-training TI was 22% (147/663), which was significantly higher than the 6% rate observed before training (6%) (p < 0.001), but still much lower than the 82.5% (302/366) observed in the host center (82.5%).

Data were collected on 80.5% of the patients from the host center (243/302) and on 85% (125/147) from the home center. While average age was higher in women undergoing laparoscopic hysterectomy at the host center (P < 0.001), parity rate (P = 0.023), number of c-sections (P < 0.001), and overweight rate (P = 0.020) were higher among those operated at the home center. Clinical parameters did not significantly differ between centers (Table 1).

**Table 1:** Epidemiological and clinical data of patients undergoing laparoscopic hysterectomy at Botucatu Medical School Hospital (FMB)/Universidade Estadual Paulista “Júlio de Mesquita Filho” (UNESP) (n = 125) and at Clermont-Ferrand Hospital and University Center (CHU) – Estaing (n = 243).

Variable	FMB/UNESP	CHU – Estaing	P-value*
Age (years)^a^	45 (28; 74)	51 (29; 85)	< 0.001
Parity^a^	3 (0; 7)	2 (0; 8)	0.023
C-section^a^	1 (0; 4)	0 (0; 3)	< 0.001
BMI (Kg/m^2^)^b^	28.94 (± 5.11)	26.40 (±7,10)	0.020
Hypertension^c^	30 (24%)	57 (23.5%)	> 0.05
*Diabetes melitus* c	6 (4.8%)	13 (5.4%)	> 0.05
Smoking^c^	14 (11.2%)	33 (13.6%)	> 0.05
Use of HT^c^	4 (3.2%)	16 (6.6%)	> 0.05
Breast cancer^c^	4 (3.2%)	3 (1.2%)	> 0.05

a Median; minimum and maximum values in parentheses;

b average value; standard deviation in parentheses;

c percentage distribution n (%);

* significant difference if p < 0.05;

C-section: caesarean section; BMI: body mass index; HT: hormonal therapy. Source: Authors.

Uterine fibroids was the most frequent reason for laparoscopic hysterectomy in both centers (Table 2). Laparoscopic hysterectomy was more frequently performed for uterine prolapse repair and for cancer treatment in the host center (Table 2).

**Table 2:** Principal indications for laparoscopic hysterectomy at Botucatu Medical School Hospital (FMB)/Universidade Estadual Paulista “Júlio de Mesquita Filho” (UNESP) (n = 125) and at Clermont-Ferrand Hospital and University Center (CHU) – Estaing (n = 243).

Indication	FMB/UNESP (%)	CHU – Estaing (%)	P-value*
Unspecified bleeding^a^	8 (6.4)	9 (3.7)	> 0.05
Uterine fibroid^a^	56 (44.8)	73 (30)	> 0.05
Adnomyosis^a^	18 (14.4)	24 (9.9)	> 0.05
Prolapse^a^	2 (1.6)	46 (18.9)	< 0.05
Chronic pelvic pain^a^	2 (1.6)	8 (3.3)	> 0.05
Endometriosis^a^	10 (8)	14 (5.8)	> 0.05
Adnexal/abdominal mass^a^	8 (6.4)	10 (4.1)	> 0.05
Oncologie^a^	21 (16.8)	59 (24.3)	< 0.05

a Percentage distribution n (%);

* significant difference if p < 0.05. Source: Authors.

There were no statistical differences in length of hospital stay, preoperative uterine volume, and surgical specimen weight (Table 3). However, significantly shorter mean operative time and lower blood loss during the procedure were observed in the host center group (Table 3).

During the study period, the rate of reoperations was 2% at the host center, whereas no reoperation was observed at the home center (Table 3).

**Table 3:** Data on laparoscopic hysterectomy procedure at Botucatu Medical School Hospital (FMB)/Universidade Estadual Paulista “Júlio de Mesquita Filho” (UNESP) (n = 125) and at Clermont-Ferrand Hospital and University Center (CHU) – Estaing (n = 243).

Variable	FMB/UNESP	CHU – Estaing	P-value*
Uterine volume in cm^3^ (imaging)^a^	172.62 (± 108.16)	247.67 (± 337.22)	0.818
Days of hospitalization^b^	3 (1; 8)	3 (2; 12)	0.728
Operative time (minutes)^a^	179.76 (± 58.19)	145.21 (± 62.84)	< 0.001
Estimated blood loss (mL)^c^			< 0.001
< 50	31 (24.8%)	236 (97.2%)	
< 200	72 (57.6%)	5 (2%)	
200 – 500	18 (14.4%)	0 (0%)	
> 500	4 (3.2%)	2 (0,8%)	
Surgical specimen weight (g)^a^	183.41 (± 93.44)	181.19 (± 196.48)	0.09
Complications rates^c^	5 (4%)	11 (4.5%)	> 0.05
Reintervention^c^	0 (0%)	5 (2%)	> 0.05

a Average value; standard deviation in parentheses;

b median; minimum and maximum values in parentheses;

c percentage distribution n (%);

* significant difference if p < 0.05;

imaging: ultrasound, computed tomography, magnetic resonance imaging. Source: Authors.

## Discussion

This study demonstrated that fellowship training in a world-renowned specialized center allowed standardizing the surgical steps of laparoscopic hysterectomy in the home center and thus significantly improved its TI in four years, with comparable surgical outcomes.

**Figure 1:**
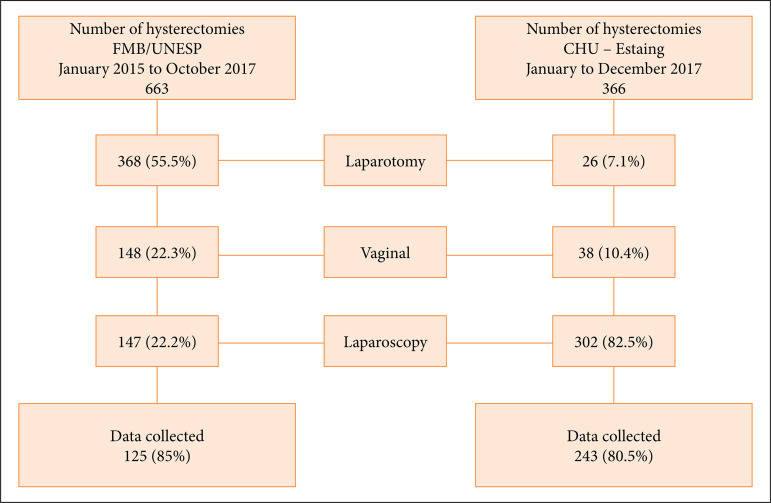
Distribution of hysterectomies by technical approach by institution.

FMB: Botucatu Medical School Hospital; UNESP: Universidade Estadual Paulista “Júlio de Mesquita Filho”; CHU – Estaing: Clermont-Ferrand Hospital and University Center. Source: Authors.

The host center is a world reference center, regarded as one of the birthplaces of modern laparoscopy, with a TI of 90%, a significant accomplishment when compared with the rate of the home center that is still under development^15^,^22^. Moreover, it is worth of note that, although TI calculation is most based on both vaginal and laparoscopic hysterectomies, only laparoscopic hysterectomies were included in our analysis in order to demonstrate the effect of training (otherwise, overall TI was 92.9% in the host center and 44.5% in the home center during the study period) (Fig. 1). Nonetheless, the considerable improvement observed in the home center’s TI during the study period clearly indicates that standardizing both the technique and the teaching process can effectively improve performance.

The TI was devised based on the evidence that vaginal and laparoscopic hysterectomy result in less complications and better outcomes when compared with the abdominal approach. To compare approaches to hysterectomy, several studies have focused on duration of surgery, length of stay in hospital, complication rate, hospital cost, and quality of life^15^,^23^-^27^.

Comparison between study centers showed that operative time was significantly faster, and blood loss during surgery was significantly lower in the host center, while length of hospital stay and complication rate were similar between centers.

These data show that the technique for high complexity procedures is better established in the host center, where laparoscopic hysterectomy for uterine prolapse repair and for cancer treatment was more frequent. Indeed, more complex cases (such as patients with cancer or severe endometriosis) have been regularly managed in the host centers for decades whereas in the home center oncological laparoscopic surgeries were started only in 2016 and laparoscopic prolapse repair in 2017^28^. This can also explain why the rate of reoperations during the study period was 2% at the host center, whereas no reoperation was observed at the home center.

Notably, the analysis of further and longer lasting effects of laparoscopic gynecological surgery training on the home center performance had to be postponed due to the COVID-19 pandemic, but it shall be resumed in the near future.

## Conclusion

Taken together, our results demonstrate that access to high-quality laparoscopic training opportunities in highly experienced reference centers can have substantial benefits. It fosters the professional growth of individual surgeons, promotes high standards and best practices, and favors the structuring and development of new surgical care and training centers in the recipients’ communities, especially in developing countries. Such was the case of the experience described herein, which allowed the home center to increase their TI based on laparoscopic procedures from 6 to 22% over a period of four years.

## Data Availability

All data sets were generated or analyzed in the current study.
